# Stretchable and Skin‐Attachable Electronic Device for Remotely Controlled Wearable Cancer Therapy

**DOI:** 10.1002/advs.202205343

**Published:** 2023-02-02

**Authors:** Xiaohui Ma, Xiaotong Wu, Shitai Cao, Yinfeng Zhao, Yong Lin, Yurui Xu, Xinghai Ning, Desheng Kong

**Affiliations:** ^1^ College of Engineering and Applied Sciences Jiangsu Key Laboratory of Artificial Functional Materials Nanjing University Nanjing 210046 China; ^2^ State Key Laboratory of Analytical Chemistry for Life Science Nanjing University Nanjing 210046 China; ^3^ National Laboratory of Solid State Microstructure Collaborative Innovation Center of Advanced Microstructures Chemistry and Biomedicine Innovation Center Nanjing University Nanjing 210093 China

**Keywords:** cancer therapy, liquid metal conductor, stretchable electronics, wearable heater, wearable therapy

## Abstract

Surgery represents a primary clinical treatment of solid tumors. The high risk of local relapse typically requires frequent hospital visits for postoperative adjuvant therapy. Here, device designs and system integration of a stretchable electronic device for wearable cancer treatment are presented. The soft electronic patch harnesses compliant materials to achieve conformal and stable attachment to the surgical wound. A composite nanotextile dressing is laminated to the electronic patch to allow the on‐demand release of anticancer drugs under electro‐thermal actuation. An additional flexible circuit and a compact battery complete an untethered wearable system to execute remote therapeutic commands from a smartphone. The successful implementation of combined chemothermotherapy to inhibit tumor recurrence demonstrates the promising potential of stretchable electronics for advanced wearable therapies without interfering with daily activities.

## Graphical abstract

A stretchable electronic patch is integrated into a self‐powered platform for skin‐attached administration of synergistic chemothermotherapy under remote commands from a smartphone. The successful inhibition of postoperative recurrence demonstrates the promising potential of stretchable electronic devices for wearable cancer treatment without interfering with daily activities.

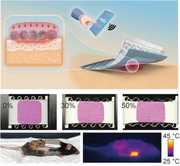

## 1 Introduction

Cancer is among the leading causes of death worldwide to reduce life expectancy.^[^ ^1^ ^]^ Surgical recession represents a commonly adopted clinical approach for treating solid tumors.^[^ ^2^ ^]^ Local relapse and distant metastasis are often encountered in postoperative recovery to affect the treatment outcome.^[^ ^3^ ^]^ Some residual tumor cells are inevitably present in the wound tissue due to their infiltrative and invasive properties.^[^ ^4^ ^]^ Chemotherapy is employed as an indispensable postoperative adjuvant treatment to reduce recurrence rates.^[^ ^5^ ^]^ To minimize the unpleasant side effects, photothermal therapy in combination with chemotherapy is often deployed as a promising strategy for synergistic therapeutic effects.^[^ ^6^ ^]^ The photothermal conversion of nanoagents under near‐infrared radiations induces local hyperthermia for suppressed cancer cell growth and improved drug sensitivity.^[^ ^7^ ^]^ In spite of the enhanced effectiveness, the widespread clinical adoption of photothermal therapy is hindered by several stringent requirements on frequent administration of photothermal nanoagents, capital investment in high‐intensity lasers, and a well‐controlled hospital environment.^[^ ^6b^ ^]^

Wearable devices offer an alternative platform for medical therapy without interfering with daily activities. Conventional heat packs and wraps for thermal treatment exhibit rigid and bulky form factors, which represent practical challenges for the efficient and reliable delivery of thermal energy to the human body.^[^ ^8^ ^]^ To overcome the limitation, stretchable electronics have emerged as a disruptive technology featuring compliant material components and excellent mechanical deformability.^[^ ^9^ ^]^ The conformal integration of functional devices with the curvilinear human body allows the stable acquisition of health‐related physical parameters and biochemical markers.^[^ ^10^ ^]^ The intimate skin‐device interface also provides the basis for wearable therapy based on thermal actuation, electrical stimulations, and controlled drug release, thereby enabling promising applications in pain relief, diabetes management, and accelerated wound healing.^[^ ^11^ ^]^ The combination with energy conversion/storage devices further enables self‐powered untethered systems for wearable and implantable applications.^[^ ^12^ ^]^ In spite of the progress, stretchable electronics have limited implementations in wearable cancer treatment. Some recent attempts have demonstrated the promising potential of electro‐thermal actuation to inhibit cancer growth.^[^ ^13^ ^]^ An effective approach to suppress postoperative recurrence still requires additional developments in stretchable electronics to execute the advanced therapeutic protocol.

In this study, we report the development of a stretchable electronic patch coupled with a supporting system for remotely controlled wearable cancer treatment. The electronic patch is composed of a soft composite nanotextile and a compliant electroresistive heater to achieve conformal lamination on the surgical site. The liquid metal conductor in serpentine mesh design allows the heater to retain a fairly stable temperature upon tensile stretching. The phase‐change microcarriers embedded in the nanotextile dressing allow on‐demand anticancer drug release under electro‐thermal actuation. An additional flexible control circuit and compact battery complete a fully untethered system that executes therapeutic commands remotely sent from a smartphone. The stretchable electronic patch establishes a long‐term reliable interface with the surgical wound for postoperative treatment involving combined electrical hyperthermia and programmed drug dosage. The effective suppression of tumor recurrence demonstrates the promising potential of the stretchable electronic platform for advanced wearable cancer therapy.

## 2 Results and Discussion

### 2.1 Design of Stretchable and Skin‐Attachable Electronic Patch

**Figure 1:**
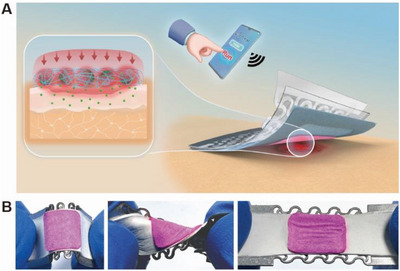
Intrinsically stretchable electronic patch for wearable cancer therapy. A) Schematic illustration of the conformal attachment of the stretchable electronic patch to the postoperative surgical wound for on‐demand delivery of therapeutic drugs and thermal actuation under remote commands from a smartphone. B) As‐prepared electronic patch under different mechanical manipulations, including bending (left), twisting (middle), and stretching (right).

As schematically illustrated in **Figure** **1**A, a stretchable electronic patch comprising an electroresistive heater and a composite nanotextile dressing is conformally mounted on a postoperative wound site of human skin. A temporary thermal actuation is generated by the electronic patch to induce local hyperthermia under the control command from a smartphone. The anticancer drug encapsulated in thermo‐responsive microcarriers is efficiently released into the tissues at elevated temperatures and then largely suppressed after cooling to the body temperature. A wearable platform of combined cancer therapy has been established to allow flexible control over dosage and frequency under remote commands. As shown in Figure 1B, the as‐prepared electronic patch is mechanically compliant and sufficiently durable to withstand various manipulations in terms of twisting, bending, and stretching. The deformable form factor is essential to achieve intimate interactions with soft and curvilinear biological tissues, thereby providing a stable interface for various wearable therapies.

### 2.2 Compliant Dressing of Composite Nanofiber Textile

**Figure 2:**
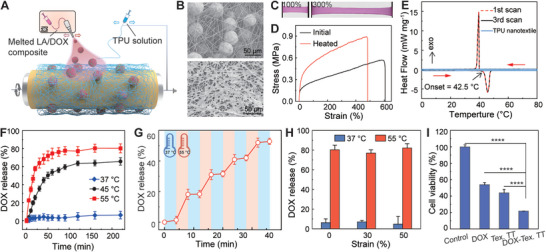
Compliant composite nanotextile dressing. A) Schematic illustration of the co‐assembly process to create composite nanofiber textile. B) SEM images of the composite nanotextile at the pristine state (top) and after thermal treatment (bottom). C) Optical images of the RhB‐loaded composite nanotextile at different tensile strains. D) Uniaxial stress‐strain curves of the composite nanotextile at the pristine state and after thermal treatment. E) Differential scanning calorimetry (DSC) curves showing the stable phase transitions of trapped microcarriers at multiple scans. F) DOX release profile of the composite nanotextile at different temperatures. G) Release profile in response to thermal actuation cycles. H) Cumulative release of DOX from composite nanotextiles within ≈4 h at 37 and 55 °C under different tensile strains. I) Cell viability of B16F10 cells exposed to the pristine culture medium (control), free DOX (0.5 µg mL^−1^), TPU nanotextile at 55 °C for 5 min (Tex. TT), and composite nanotextile at 55 °C for 5 min (DOX‐Tex. TT). Data are presented as mean ± SD of *n* = 6. Statistical differences are determined by two‐tailed Student's *t* tests (*p < 0.05, **p < 0.01, ***p < 0.001, and ****p < 0.0001).

Compliant wound dressing is an indispensable component of postoperative wound management. According to the schematic illustration in **Figure** **2**A, a co‐assembly technique is established here to create a composite nanofiber textile as a wound dressing. Briefly, a biocompatible TPU elastomer is dissolved in an organic solvent and electrostatically drawn into nanoscale fibers based on the electrospinning technique. A biobased phase‐change material of lauric acid (LA) is selected as the organic matrix to encapsulate anticancer drugs.^[^ ^14^ ^]^ After melting at elevated temperature, the mixture is fed into an ultrasonic spray nozzle and then atomized into relatively uniform microparticles of 44.4 ± 8.0 µm, as shown in Figure S1 (Supporting Information). All in situ generated nanofibers and microparticles are assembled into a composite nanotextile on the collector. A variety of drugs are readily loaded in the phase change microcarriers and fully embedded into the composite nanotextile, as shown in Figure S2 (Supporting Information). In Figure 2B, the SEM image reveals the porous microstructure of the composite nanotextile containing plenty of microcarriers. In spite of the initial high contact angle, the nanotextile gradually converts into a superhydrophilic material to allow complete uptake of wound exudate, as shown in Figure S3 (Supporting Information). Individual microparticles are physically trapped between TPU nanofibers as a result of the co‐assembly process, thereby preventing their fast clearance from the wound site. As‐prepared composite nanotextile is highly stretchable by using TPU elastomer to create the nanofibers (see Figure 2C). In Figure 2D, the corresponding stress‐strain curve demonstrates the compliant mechanical properties with a low modulus of ≈1.6 MPa and a large fracture strain of ≈600%. According to DSC thermograms in Figure 2E, the activation temperature of the composite nanotextile is at ≈42.5 °C to trigger the onset of melting of the microcarriers. After a temperature sweep, the microcarriers are fully melted and then resolidified into irregular flakes, as shown in Figure 2B. Despite the notable changes in the morphology, the composite nanotextile still preserves the highly porous microstructure and excellent stretchability up to ≈480% strain (see Figure 2D). In addition, the identical DSC thermograms in multiple scans suggest reliable phase‐change characteristics for repeated dosing (see Figure 2E).

The release kinetics of the composite nanotextile dressing are systematically investigated for controlled drug delivery. Figure 2F shows the release profile of DOX into PBS solution at different temperatures. A negligible release of ≈6.1 ± 4.2% within 4 h at the normal body temperature is ascribed to the highly hydrophobic and slow hydrolytic characteristics of the lauric acid matrix. In comparison, the cumulative release is 65.7 ± 4.1% at 45 °C and 80.3 ± 5.0% at 55 °C due to the melting of phase‐change microcarriers (see Figure 2F and Figure S4, Supporting Information). In these activated conditions, the elevated temperature promotes the increased release by accelerating the molecular diffusions.^[^ ^14^, ^15^ ^]^ In Figure 2G, a stepwise drug release profile is achieved by repetitive thermal actuation involving rapid release at the elevated temperature and negligible leakage at the normal body temperature. A typical composite nanotextile supports repeated thermal activations with fairly stable dosing for at least four cycles. In addition, the drug release behaviors at 37 and 55 °C are negligibly influenced by tensile deformations of up to 50% strain (see Figure 2H and Figure S5, Supporting Information). Reliable dose control irrespective of mechanical manipulations is essential for the practical implementation of wearable therapy.

In Figure S6 (Supporting Information), the cell viability analysis confirms the excellent biocompatibility of the composite nanotextile utilizing benign materials components. Systematic cytotoxicity tests with B16F10 tumor cells further reveal the therapeutic potentials of the drug‐loaded nanotextile dressing. In Figure 2I, the cell viability is 54.0 ± 2.5% with the addition of free DOX at 0.5 µg mL^−1^ and 44.0 ± 4.1% with a short exposure to hot PBS solution at 55 °C. In Figure S7 (Supporting Information), the elevated temperature imposes a universal inhibition effect on different cell types consistent with previous reports.^[^ ^16^ ^]^ In the case of 55 °C exposure, the tumor cells are slightly more sensitive than normal cells to justify the choice of the treatment temperature. A substantial drop in the survival rate to 21.8 ± 0.02% is associated with the DOX‐loaded composite nanotextile after thermal activation in 55 °C PBS solution. In addition, the live/dead cell staining further confirms the highest cell mortality of thermally activated composite nanotextile, as shown in Figure S8 (Supporting Information). The synergistic inhibitory effects of combined thermal and chemical treatments are ascribed to cytotoxic heat exposure, accelerated drug release, and improved cell membrane permeability.^[^ ^6^, ^17^ ^]^

### 2.3 Compliant Electroresistive Heater

**Figure 3:**
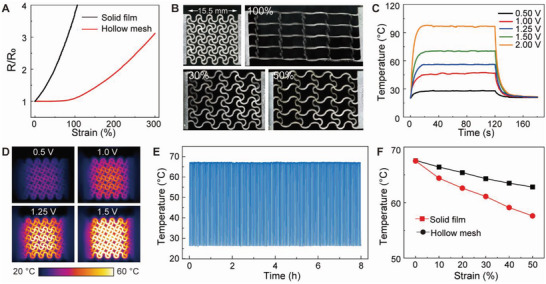
Compliant electroresistive heater. A) Normalized resistance versus tensile strain of liquid metal conductors. B) Liquid metal‐based serpentine mesh heater at different tensile strains. C) Temperature profiles at different applied voltages. D) Infrared camera images of the heater at different voltages. E) Temperature responses to 120 on/off voltage cycles with an amplitude of 1.5 V. F) Temperature as a function of the tensile strain under a drive voltage of 1.5 V.

The compliant electroresistive heater is essential for the reliable delivery of thermal actuation. Intrinsically stretchable conductors represent the key building materials to determine the overall performance of soft electronic devices. A eutectic gallium indium (EGaIn) alloy, commonly termed liquid metal, is an attractive candidate material featuring excellent electronic conductivity (3.4 × 10^4^ S cm^−1^), fluid‐like deformability, and excellent biocompatibility.^[^ ^18^ ^]^ A Cu/Cr thin film is thermally evaporated onto the TPU substrate, as the wetting layer to enable adhesion with the bulk form of nonoxidized liquid metal. A smooth and uniform liquid metal film is obtained by removing extra EGaIn alloy through high‐speed rotation in a spin coater. The resistance of the liquid metal film is highly sensitive to tensile strains, as shown in **Figure** **3**A. The composite is further engraved into a hollow serpentine mesh based on selective laser ablation.^[^ ^19^ ^]^ The TPU substrate has been incorporated with charcoal powders for enhanced optical absorption across visible to near‐infrared regions, as shown in Figure S9A (Supporting Information). Arbitrary features are generated in a subtractive mode by steering the laser spot across the sample along programmed paths. A single laser pass efficiently removes a ≈90 µm thick layer through heat‐induced vaporization, as shown in Figure S9B (Supporting Information).

In Figure 3B, the serpentine mesh design of the liquid metal conductor achieves minor resistance changes upon stretching by dissipating tensile deformations through bending‐controlled motions.^[^ ^19b^ ^]^ Specifically, the resistance increases by ≈3% at 100% tensile strain. In addition, the serpentine mesh also demonstrates durable mechanical properties during strain‐controlled fatigue tests, as shown in Figure S10 (Supporting Information). In Figure 3C, the temperature can rapidly rise from room temperature to ≈67 °C at an applied voltage of 1.5 V due to the excellent conductivity. The low operating voltages are essential to alleviate safety concerns of skin‐attachable devices.^[^ ^20^ ^]^ In Figure S11 (Supporting Information), the temperature shows minor fluctuations within 0.5 °C at a fixed voltage, enabling precise control by simply adjusting the operating voltages. According to the thermal image in Figure 3D, homogenous temperature distribution at the heating center suggests the uniform electrical properties of the serpentine mesh heater. The temperature distribution is highly consistent with finite element analysis, as shown in Figure S12 (Supporting Information). The simulation also allows the reliable prediction of the steady‐state temperature at each driving voltage. The compliant heater is further evaluated by 120 on/off thermal actuation cycles, as shown in Figure 3E. The stable temperature responses demonstrate the robust performance of the electroresistive heater for repetitive operations. In response to 50% tensile strain, the heater exhibits a minor drop in the surface temperature by 4.8 °C, as shown in Figure 3F, primarily due to the expansion of the heating area.^[^ ^19b^ ^]^ The strain‐insensitive heating property is beneficial from the stable resistance to allow the reliable delivery of thermal actuation. In contrast, the liquid metal film shows a notable change of 9.9 °C under the same strain level. The serpentine mesh structure also improves mechanical compliance with a low modulus of 41.0 kPa, as shown in Figure S13 (Supporting Information).

### 2.4 Fabrication and System Integration of Stretchable Electronic Patch

**Figure 4:**
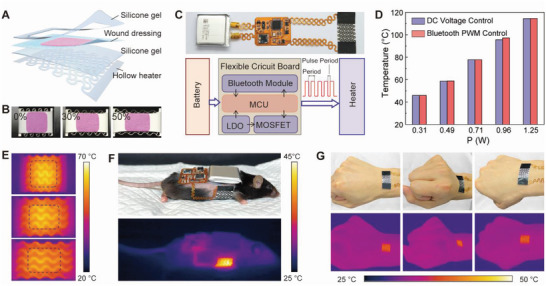
Integrated self‐powered wearable electronic system. A) Schematic showing the layer‐by‐layer construction of the stretchable electronic patch. B) Optical images of the patch at different strains. C) Optical image of a fully integrated electronic system (top) and block diagram of the operation principles (bottom). D) Temperature of the electronic patch versus electrical power under different driving modes. E) Infrared camera images of the electronic patch at 0% (top), 30% (middle), and 50% (bottom) strains. The dashed line indicates the area of composite nanotextile loaded with anticancer drugs. F) Optical image (top) and corresponding infrared camera image (bottom) of a fully integrated electronic system operated on a freely moving mouse. G) Optical images (top) and corresponding infrared camera images (bottom) showing the stable operation of the integrated system attached to the forearm under hand motions.

A stretchable electronic patch is constructed in a layer‐wise sequence, as schematically illustrated in **Figure** **4**A. The composite nanotextile is bonded to the electroresistive heater with silicone gel adhesive. An additional layer of silicone gel is applied to the edge of the patch for conformal attachment to the body. The approach allows the facile fabrication of electronic patches with different sizes, as shown in Figure S14 (Supporting Information). In Figure 4B, a representative electronic patch exhibits compliant mechanical properties to withstand tensile deformations of up to 50% strain. A stretchable electronic patch is coupled with a flexible integrated circuit and a compact lithium‐ion battery to form a self‐powered electronic system, as shown in Figure 4C. The specific design layout and mechanical flexibility demonstration of the integrated circuit are shown in Figures S15 and S16 (Supporting Information). The serpentine interconnects are introduced fordeformability on the system level.^[^ ^12^, ^21^ ^]^ Figure 4C also provides a schematic diagram to outline the operation principle of the system. A microcontroller unit (MCU) is installed in the flexible circuit to execute control commands remotely sent from smartphones via Bluetooth protocol. Pulse width modulation (PWM) on a transistor switch allows the convenient adjustment of the duty ratio and hence the heating power.^[^ ^20^ ^]^ In spite of different operation modes, the electronic patch achieves consistent temperatures due to the Joule heating mechanism (Figure 4D). The steady‐state temperature is linearly correlated with the supplied electrical power (see Figure S17, Supporting Information). According to infrared camera images in Figure 4E, the drug‐loaded area is inside the uniform heating zone of the electronic patch to ensure reliable control over the drug release profile. A slightly modified circuit layout generated a tailored system design to match the body size of mice (see Figure S18, Supporting Information). The soft and lightweight system is carried by a freely moving mouse with the electronic patch conformally attached to the skin, as shown in Figure 4F and Video S1 (Supporting Information). A compact battery powers the system to enable untethered and remotely controlled wearable therapy. All PWM control parameters of the surface temperature have been carefully calibrated with IR images, as shown in Figure S19 (Supporting Information). Notice that the battery has extra capacity to support heating operation at ≈55 °C for 5 min corresponding to >90 thermal actuation cycles (see Figure S20, Supporting Information). A smaller battery can be used to reduce the overall form factor of the system. In addition to animal experiments, a standard electronic system is also mounted on the forearm of a human subject with the heating temperature and duration set from the smartphone, as shown in Video S2 (Supporting Information). The temperature is controlled by modulating the power supply to the heater through PWM modulation. The compliant mechanical property is essential for the stable attachment of the electronic patch to the human wrist without delamination during various deformations including stretching, compression, and twisting, as shown in Figure 4G and Video S3 (Supporting Information). The device design and system architecture, therefore, exhibit promising translational potentials.

### 2.5 In Vivo Postoperative Wearable Cancer Therapy

**Figure 5:**
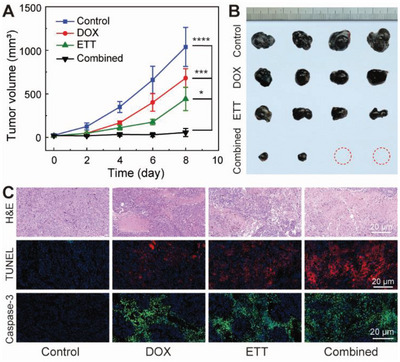
In vivo efficacy of wearable cancer therapy. A) Tumor growth curve of postoperative tumor models receiving no treatment (control), free DOX, wearable electro‐thermal therapy (ETT), and wearable combined therapy. B) Optical images of harvested tumors after the treatments. C) Histopathological analysis of tumor tissue sections based on H&E, TUNEL, and Caspase‐3 staining. Data are presented as mean ± SD of *n* = 4. Statistical differences are determined by one‐way ANOVA (*p < 0.05, **p < 0.01, ***p < 0.001, and ****p < 0.0001).

The stretchable electronic patch is deployed for wearable therapy on wound sites of postoperative tumors. In female black mice, B16F10 melanoma tumors are established in armpits and then surgically debulked by ≈2/3 to simulate nonideal surgical conditions.^[^ ^22^ ^]^ The mice are randomly divided into four groups, including i) the control group (without treatment), ii) the TPU nanotextile dressing containing free DOX group, iii) the wearable electro‐thermal therapy (ETT) group, and iv) the wearable combined therapy group. The stretchable electronic patch is conformally attached to the surgical wound site with abundant exudate for wearable treatments, as shown in Figure S21 (Supporting Information). The absorbed exudate in the nanotextile dressing provides the aqueous medium for reliable drug delivery. Mice weight and tumor volume are monitored every 2 d for a therapy duration of 8 d. The biocompatibility of the electronic patch is supported by the negligible body weight changes and toxic symptoms during the treatment (Figure S22, Supporting Information). In the control group, the recurrent melanoma tumor reaches ≈1038 mm^3^ and ≈0.64 ± 0.10 g on day 8, as shown in **Figure** **5**A and Figure S23 (Supporting Information). The average melanoma tumors on day 8 are reduced to 680 mm^3^ and 0.44 ± 0.05 g in the free DOX group and 442 mm^3^ and 0.30 ± 0.074 g in the ETT group. The ETT has a strong treatment effect on the tumor despite minor damage to the surrounding tissue, as shown in Figure S24 (Supporting Information). The suppressed thermal damage to healthy tissues is associated with their adaptive vascular system to accelerate the blood flow, reducing the temperature rise after hyperthermic stimulation.^[^ ^23^ ^]^ A further reduction of the tumor weight to 0.04 ± 0.04 g is encountered in the combined treatment group to suggest an excellent antitumor effect. Optical images of the wound sites are also captured to show the different treatment efficacy (Figure S25, Supporting Information). In Figure 5B, the harvested tumors further allow a clear comparison of the treatment approaches, thereby highlighting the pronounced inhibition effect of the combined treatment on the tumor recurrence. As shown in Figure 5C, histopathological analysis (H&E, TUNEL, and Caspase‐3 staining) of the tumor tissue suggests significant inhibition of the nuclear differentiation and increased apoptosis of tumor cells in the combined wearable treatment as compared with other groups. In Figure S26 (Supporting Information), H&E staining of major organs reveals negligible damages or inflammations as additional evidence for the excellent biosafety of these treatments. The soft electronic patch provides a powerful skin‐attachable platform of synergistic chemo‐thermotherapy to suppress postoperative recurrence. A straightforward development of this technology is direct cancer treatment utilizing transdermal patch designs. Notice that the permeation enhancer strategies are proven effective for localized drug delivery, including chemical additives, microneedle arrays, and iontophoresis modules.^[^ ^11^, ^12^, ^24^ ^]^

## 3 Conclusions

In summary, we report material developments, device designs, and system integration strategies of a stretchable and skin‐attachable electronic patch for wearable cancer treatment. An electroresistive heating element of the patch harnesses a liquid metal conductor and serpentine mesh design to retain stable temperatures upon mechanical deformations. A compliant and biocompatible composite nanotextile with embedded phase‐change microcarriers is laminated on the patch as a temperature‐responsive dressing for on‐demand drug release. The entire electronic patch is soft and stretchy to allow conformal coverage on the surgical wound, providing a long‐term stable interface for postoperative cancer treatment. An additional flexible circuit and a compact lithium‐ion battery constitute a self‐powered wearable system to achieve intimate integration with the human body, thereby enabling reliable delivery of thermal actuation and drug dosage under commands from a smartphone. The successful inhibition of recurrent tumor growth from in vivo experiments demonstrates the promising translational potential of stretchable electronics for advanced wearable cancer therapy.

## 4 Experimental Section

### Materials

All chemical reagents and raw materials were commercially acquired, including thermoplastic polyurethane (TPU, Tecoflex SG80A) from Lubrizol Corporation, poly(vinyl alcohol) (PVA, *M*_w_ = 67 000), and doxorubicin hydrochloride (DOX, 97%) from Shanghai Aladdin Bio‐Chem Technology Co., Ltd., lauric acid (LA, 98%) and Rhodamine B (RhB) from Heowns Biochemical Technology Co., Ltd., silicone gel (Silbione RT GEL 4645A/B) from Elkem Silicones, methyl thiazolyl tetrazolium (MTT) from Beyotime Institute of Biotechnology Co., Ltd., phosphate‐buffered saline (PBS), Roswell Park Memorial Institute (RPMI) 1640 medium, and Dulbecco's modified Eagle's medium (DMEM) from Nanjing KeyGen Biotech Co., Ltd., fetal bovine serum (FBS) from Gibco (CA), tetrahydrofuran (THF), *N*,*N*‐dimethylformamide (DMF), methanol, hydrochloric acid (HCl), dimethyl sulfoxide (DMSO), and sodium carboxymethyl cellulose (CMC‐Na, *M*_w_ = 90 000) from Shanghai Macklin Biochemical Co., Ltd. The liquid metal of eutectic gallium indium alloy (EGaIn) was obtained by melting a mixture of Ga and In at 80 °C for 2 h inside a glove box according to a weight ratio of 75.5:24.5. The phase change mixture was prepared by dissolving DOX (or RhB, 80 mg) and LA (2.4 g) in methanol (24 mL) and then evaporating the solvent under the ambient conditions.

### Preparation and Release Behavior of Composite Nanotextile Dressing

Composite nanotextile was fabricated through a co‐assembly process involving electrospinning and ultrasonic spray. A continuously rotating drum collector was employed to ensure the uniformity of the assembly process. The electrospinning nozzle and the ultrasonic spray nozzle were separately mounted onto motorized linear translation stages at a 90° with respect to the collector. TPU precursor was prepared by dissolvent the pellets at a concentration of 18% (w/w) in a mixed DMF/THF solvent (1/1 by weight). The precursor was loaded into a syringe (20 mL) and fed to the electrospinning nozzle at 120 µL min^−1^ by using a syringe pump (TYD01‐01, Lead Fluid Technology Co., Ltd.). A high voltage of 12 kV was applied to the nozzle at a fixed distance of 12 cm from the grounded collector. The phase change mixture was loaded into a syringe (5 mL) and melted at 60 °C with a heating jacket. A second syringe pump delivered the liquid‐state composite to the ultrasonic spray nozzle (DW‐F40‐60, Hangzhou Dowell Ultrasonic Technology, Co., Ltd.) at 50 µL min^−1^ for effective atomization into fine microparticles. A compliant wound dressing in the nonwoven textile form was generated by the assembly process and then cut into 10 × 10 mm^2^ rounded squares by a CO_2_ laser marking system (JW‐K40, Liaocheng Jingwei Laser Equipment Co.). An additional 300 µm‐thick layer of TPU nanofiber textile was deposited onto the composite dressing as the mechanical support. To evaluate the release behavior, DOX‐loaded composite nanotextiles were submerged in PBS solution held at 37, 45, and 55 °C, respectively. The fluorescence intensity of the released DOX was measured by using a Tecan Infinite 200 Pro microplate reader (*λ*_ex_/*λ*_em_ = 480/600 nm). In addition, a composite nanotextile was placed inside a homemade liquid chamber. The solution was periodically replaced with fresh PBS at 37 or 55 °C by using a syringe pump and a distribution valve. The solution was sampled at the end of each heating/cooling cycle to determine the released DOX. Stretched composite nanotextiles were clamped on acrylic frames and then placed inside PBS solution at controlled temperatures to assess their release behaviors under tensile strains. In 55 °C PBS solution, the cumulative DOX release for an extended duration of 140 h was defined as 100%. All spectra were acquired from multiple samples for statistical analysis (*n*  = 6).

### Fabrication of Compliant Electroresistive Heater

Bamboo charcoal powder (8000 mesh) was manually mixed with 10% (w/w) TPU solution in THF at a weight ratio of 1:50, followed by homogenization at 2000 rpm for 5 min in a planetary mixer (JF‐RVITV‐150, Shenzhen Junfeng Technology Co., Ltd.). The viscous mixture was drop cast onto a glass wafer decorated with a sacrificial CMC‐Na film to produce a stretchable substrate by removing the solvent through natural evaporation. The black TPU substrate doped with bamboo charcoal powder is a broadband absorber for efficient light‐to‐heat conversion under laser irradiation. A 10 nm/100 nm Cr/Cu thin film was deposited on the black TPU in a ZHD400 thermal evaporator from Beijing Technol Science Co., Ltd. Inside a NaOH aqueous solution of 4 w/v %, EGaIn was drop cast and adhered to the metalized substrate. A uniform layer of liquid metal was obtained by rotating the substrate in a spin coater at 2000 rpm for 60 s. An additional TPU layer was spin cast for encapsulation. A hollow mesh pattern was defined by selective laser ablation carried out on a 1064 nm benchtop laser marking system (XM‐FB30, Xianming Optoelectronics Equipment Co., Ltd.). The laser beam was controlled by a pair of galvanometric mirrors and then focused on the sample by using an f‐theta lens. Selective laser ablation was carried out with a laser power of 12 W and a scan speed of 2000 mm s^−1^. All undesired areas were subtractively removed to generate the hollow mesh by steering the laser spot according to the programmed paths. The as‐prepared heater was subsequently released from the glass wafer by dissolving the sacrificial layer in warm water.

### Characterizations

SEM characterizations were carried out by using a Zeiss Ultra55 Field Emission Scanning Electron Microscope. Optical microscopy images were acquired by a Keyence VHX‐6000 Digital Microscope. Fluorescence microscopy images were taken by a Nikon inverted fluorescence microscope and an Olympus FV3000 confocal laser scanning microscope. Optical topographic images were collected by using a Keyence KX1000 confocal laser scanning microscope. All optical images and videos were captured by a Fujifilm X‐T10 camera. A Shimadzu AGS‐X universal testing machine equipped with a 50 N load cell was employed to acquire uniaxial tensile stress‐strain curves. The resistance was measured under a four‐point configuration by using a GW Instek GOM‐805 milliohm meter. The electromechanical properties were evaluated based on a homemade motorized linear stage. DSC thermograms were acquired at a scan rate of 5 °C min^−1^ by using a DSC823e differential scanning calorimeter from Mettler Toledo Instruments (Shanghai) Co., Ltd. The surface temperature was obtained from noncontact measurements by using a Testo 885‐2 thermal imaging camera. The galvanostatic discharge of the lithium‐ion battery was performed with a CHI 660E electrochemical workstation.

### In Vitro Biocompatibility and Cytotoxicity

Mouse melanoma cell line B16F10 and human embryonic kidney cell line 293T, and Fibroblast cell line were obtained from American Type Culture Collection (ATCC). B16F10 cells were cultured in RPMI 1640 medium supplemented with 10% FBS and penicillin/streptomycin (1%, w/v). 293T cells were cultured in DMEM medium supplemented with 10% FBS and penicillin/streptomycin (1%, w/v). Fibroblast cells were cultured in DMEM medium (high glucose) supplemented with 10% FBS, 1% NEAA, and penicillin/streptomycin (1%, w/v). The viability of 293T, Fibroblast, and B16F10 cells in vitro was tested by MTT assay to evaluate the biocompatibility and cytotoxicity, respectively. 293T cells (4000 per well) were seeded and cultured overnight at 37 °C in 96‐well plates filled with DMEM supplemented with 10% FBS and 1% penicillin/streptomycin. Fibroblast cells were also seeded overnight at 37 °C in 96‐well plates filled with DMEM medium (high glucose) supplemented with 10% FBS, 1% NEAA, and penicillin/streptomycin (1%, w/v). The culture medium was subsequently replaced by 200 µL RPMI 1640 medium containing i) control group (no treatment), ii) TPU nanotextile at 55 °C for 5 min for thermal therapy (Tex. TT), iii) DOX (0.5 µg mL^−1^), and iv) DOX‐loaded composite nanotextile at 55 °C for 5 min (DOX‐Tex. TT). After 24 h incubation, MTT solution (20 µL, 5 mg mL^−1^) was added to the culture medium and incubated for 4 h, followed by removing the medium and adding DMSO (150 µL). The absorbance intensity was measured at 570 nm using a microplate reader. Similarly, the MTT assay was also performed to reveal thermal treatment effects by exposing B16F10 and Fibroblast cells to different temperatures for 5 min. In addition, the cell death was determined using the Live/Dead staining assay. B16F10 cells (2.5 × 10^5^ per well) were seeded into 24‐well plates and cultured at 37 °C for 24 h. The culture medium was then replaced by RPMI 1640 (500 µL) containing (i–iv) samples for 24 h. After incubation, B16F10 cells were washed with PBS and stained with Hoechst 33342 (2 × 10^−3^ m) and Propidium Iodide (PI, 10 × 10^−6^ m) for 10 min at 37 °C. The red fluorescence of PI (*λ*_ex_/*λ*_em_ = 550/605 nm) and blue fluorescence of Hoechst 33342 (*λ*_ex_/*λ*_em_ = 350/460 nm) images were captured by using a Zeiss L710 fluorescence microscope. All spectra were acquired from multiple samples for statistical analysis (*n*  = 6).

### Device Fabrication and System Integration of Stretchable Electronic Patch

A layer of silicone gel precursor was brush painted onto the compliant mesh heater, followed by semi‐curing for 30 min in a 50 °C oven. The composite nanotextile dressing was laminated on the heater to form the stretchable electronic patch. The edge of the patch was brush‐painted with an additional layer of silicone gel as the skin adhesive for the conformal coverage of the surgical site. A standard patch design with an active area of 10 mm × 10 mm was loaded with 1 mg DOX in total. The as‐prepared patch was attached to the serpentine interconnects of a flexible integrated circuit. The flexible circuit board was designed in EasyEDA and manufactured by Shanghai Renren Technology Co., Ltd (see Figure S16, Supporting Information). The circuit has a microcontroller unit (MCU), a Bluetooth module for wireless communication, and a metal‐oxide‐semiconductor field‐effect transistor (MOSFET) for the solid‐state switch (see Table S1, Supporting Information). A compact lithium‐ion battery was acquired from Ampris Technologies, Inc. to power the devices in a fully untethered electronic system. Notably, the circuit layouts are slightly varied to fulfill the application settings. The patch, flexible circuit, and lithium‐ion battery form a circular arrangement to match the body size of the mouse. A linear arrangement of these components is employed to fit the human forearm. In operation, all control commands were wirelessly sent from a smartphone via Bluetooth Low Energy protocol. The microcontroller unit regulated the effective electrical power applied on the heater through the pulse width modulation (PWM) of the transistor switch. In this controlling mode, the maximal power supply is discretized by a pre‐scaler of 256 to achieve a temperature resolution better than 1 °C (see details in Figure S17, Supporting Information).

### In Vivo Cancer Treatment of Stretchable Electronic Patch System

All animal protocols were approved by the Animal Care and Use Committee of Nanjing University and conformed to the Guidelines for the Care and Use of Laboratory Animals published by the National Institutes of Health. The number of animal use permits was SYXK(Su)2019‐0056. C57BL/6J mice (female, 18–20 g) were purchased from Skbex Biotechnology. The mice were subcutaneously injected into the axilla with 2 × 10^6^ B16F10 cells. All tumors were debulked by ≈2/3 after reaching ≈65 mm^3^ to simulate nonideal surgical intervention. The postoperative mice were randomized into four experimental groups (*n* = 4 in each group): i) control group (with no treatment), ii) chemotherapy group (DOX) with TPU nanotextile containing free DOX, iii) wearable electrical thermotherapy group (ETT), and iv) wearable combined therapy group. Notice that the postoperative tumor wound has abundant exudate. The stretchable electronic patch is conformally mounted on the surgical site to uptake the exudate as the medium for drug delivery. In the ETT group, the pristine TPU nanotextile simply served as a synthetic dressing to promote wound healing. In the combined therapy group, the composite nanotextile containing DOX‐loaded microcarriers provided the basis for the triggered release of anticancer drug. In ETT and combined therapy group, the treatment was periodically administered every other day involving a single pulsed thermal actuation by heating up the patch to 55 °C for 5 min. The DOX release is 100 to 120 µg in a single thermal actuation of the combined therapy. The recurrent tumor volume and the mouse body weight were measured once every two days. The mice were sacrificed at the end of the 8‐day treatment period. The tumor tissues were dissected, weighed, photographed, and fixed with 4% paraformaldehyde. The tumor tissue sections were analyzed by hematoxylin and eosin (H&E) staining, TdT‐dependent dUTP‐biotin nick end labeling (TUNEL), and caspase‐3 staining. The major organs and tumor tissues were fixed with 4% paraformaldehyde and analyzed by H&E staining.

### Statistical Analysis

All statistical analyses were accomplished with GraphPad Prism 9 (San Diego, CA). Data were expressed as mean ± standard deviation. Differences were statistically analyzed by using an unpaired Student's *t* test or one‐way analysis of variance (ANOVA). Significance marks were categorized with the following *p* values: *p < 0.05, **p < 0.01, ***p < 0.001, and ****p < 0.0001).

## Conflict of Interest

The authors declare no conflict of interest.

## Author Contributions

D.K. and X.N. conceived the experiments. X.M. performed material preparations, device fabrications, and characterizations. X.W., X.M., Y.X., and Y.Z. performed the in vitro and in vivo experiments. S.C. assembled the flexible circuit board and developed the control programs. X.M. and D.K. wrote the original manuscript. All authors contributed to the scientific planning and discussions.

## Supporting information

Supporting Information

Supplemental Video 1

Supplemental Video 2

Supplemental Video 3

## Data Availability

The data that support the findings of this study are available in the supplementary material of this article.
