# Supplementary material for: Stretchable and Skin‐Attachable Electronic Device for Remotely Controlled Wearable Cancer Therapy
Source: Adv Sci (Weinh). 2023 Feb 2;10(10):2205343. doi: 10.1002/advs.202205343 (PMC10074095; doi:10.1002/advs.202205343)
Supplement: Supplementary file 1 — Supporting Information [file ADVS-10-2205343-s003.pdf]

ADVANCED SCIENCE
Open Access

Supporting Information

for *Adv. Sci.*, DOI 10.1002/advs.202205343

Stretchable and Skin-Attachable Electronic Device for Remotely Controlled Wearable Cancer Therapy

Xiaohui Ma, Xiaotong Wu, Shitai Cao, Yinfeng Zhao, Yong Lin, Yurui Xu, Xinghai Ning* and Desheng Kong**

Supporting Information

Stretchable and Skin-attachable Electronic Device for Remotely Controlled Wearable Cancer Therapy

Xiaohui Ma, Xiaotong Wu, Shitai Cao, Yinfeng Zhao, Yong Lin, Yurui Xu, Xinghai Ning*, Desheng Kong**

Supporting Figures

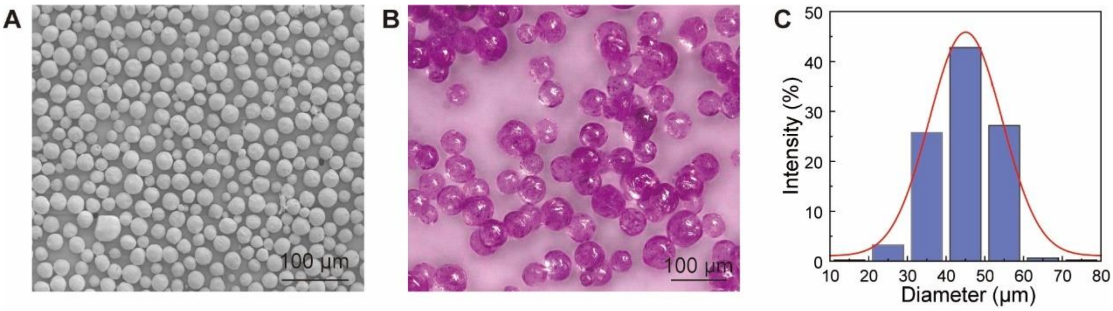

Figure S1. Characterizations of as-synthesized LA microcarriers. (A) SEM image of RhB-loaded LA microcarriers in spherical shapes. (B) Corresponding optical microscopy image revealing the characteristic reddish-purple color of RhB. (C) Histogram of size distribution with a Gaussian fit to yield the characteristic diameter of ~44.4 ± 8.0 µm.

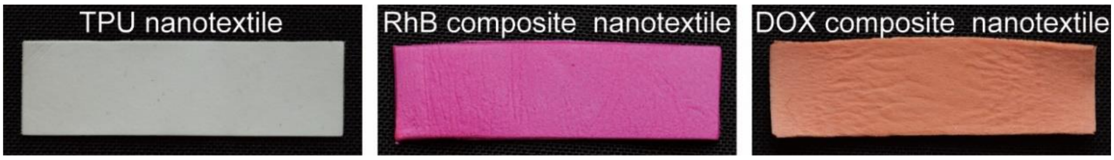

Figure S2. Optical images of TPU nanotextile and RhB/DOX-loaded composite nanotextile.

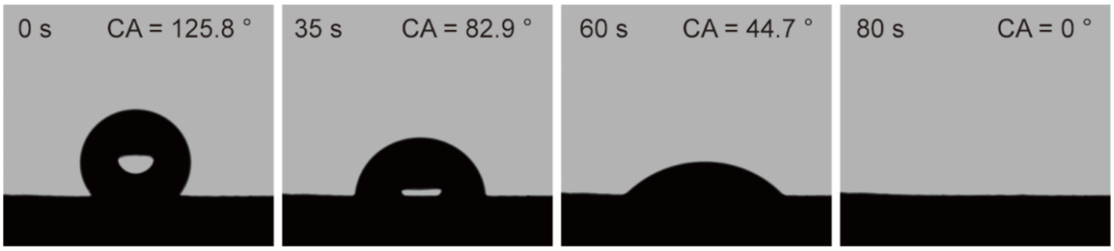

Figure S3. Dynamic wetting process of water droplet on composite nanotextile. In spite of the initial high contact angle, the nanotextile exhibits gradually improved hydrophilicity for full water absorption likely due to the hygroscopic nature of TPU elastomer.

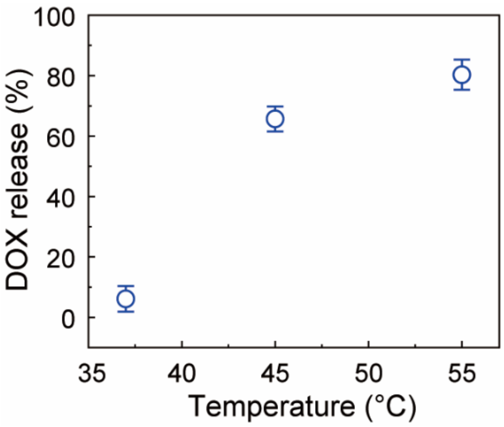

Figure S4. Cumulative DOX release from the composite nanotextile within ~4 h at different temperatures. Data are presented as mean ± SD of n = 6.

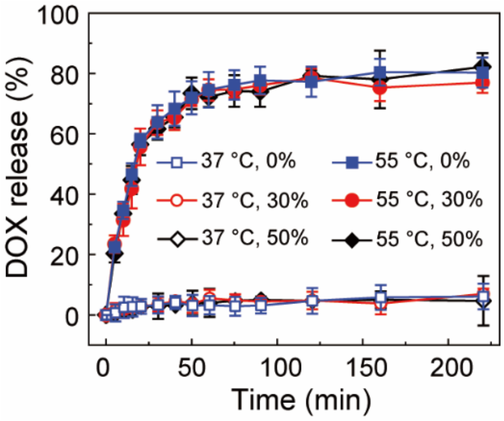

Figure S5. DOX release profile from the composite nanotextile under different tensile strains. Inside 37 and 55 °C PBS solutions, the release profiles are negligibly affected by tensile deformations of up to 50% strain. Data are presented as mean ± SD of n = 6.

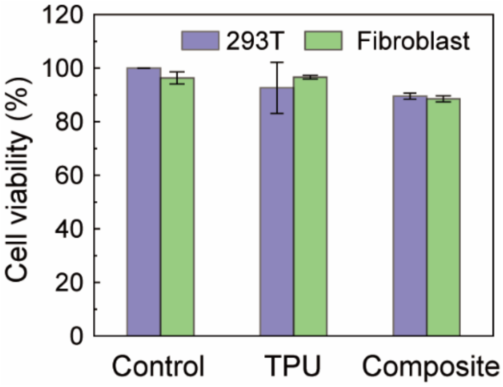

Figure S6. *In vitro* biocompatible tests. Cell viability of 293T and Fibroblast after 24 h-incubation with different samples, including normal medium (control), TPU nanotextile, and composite nanotextile. Notice that the microcarriers of the composite nanotextile do not contain any anticancer drugs. Data are presented as mean ± SD of n = 6.

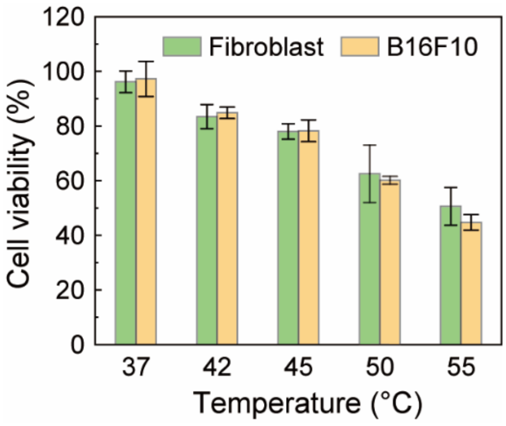

Figure S7. Cell viability of thermal treatment at different temperatures. The B16F10 and Fibroblast cells were incubated with composite nanotextile and then exposed to different temperatures for 5 min. The cell viability was analyzed by MTT assay after addition incubation for 24 h. In the case of 55 °C treatment, the tumor cells are slightly more sensitive to heat exposure than normal cells. Data are presented as mean ± SD of $n = 6$.

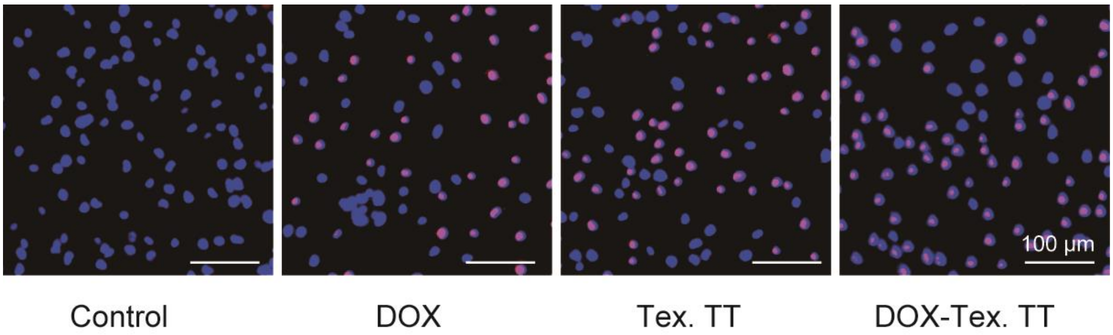

Figure S8. Fluorescent microscopy images from a live/dead (blue/purple) assay of B16F10 cells. Scale bar: 100 μm. The cells are exposed to normal medium (control), free DOX (0.5 μg mL^{-1}), TPU nanotextile at 55 °C for 5 min (Tex. TT), and DOX-loaded composite nanotextile at 55 °C for 5 min (DOX-Tex. TT).

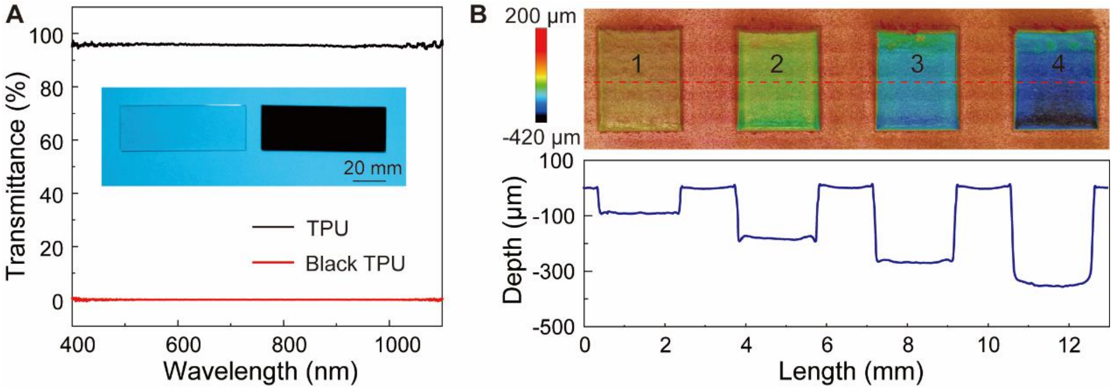

Figure S9. (A) Transmittance spectra of a standard TPU substrate and a black TPU substrate doped with bamboo charcoal powders. Inset: Corresponding optical images of the two substrates. (B) Optical topographical image (top) and height profile corresponding to the red dashed line (bottom) of an array of rectangular grooves fabricated over a black TPU substrate by selective laser ablation with different numbers of laser passes (labelled in black).

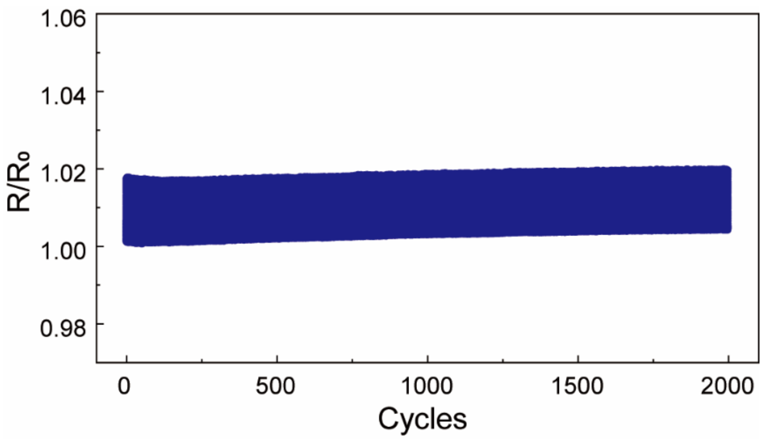

Figure S10. Electromechanical durability of the compliant heater. Change in the normalized resistance during 2000 stretch-relaxation cycles to 50% strain.

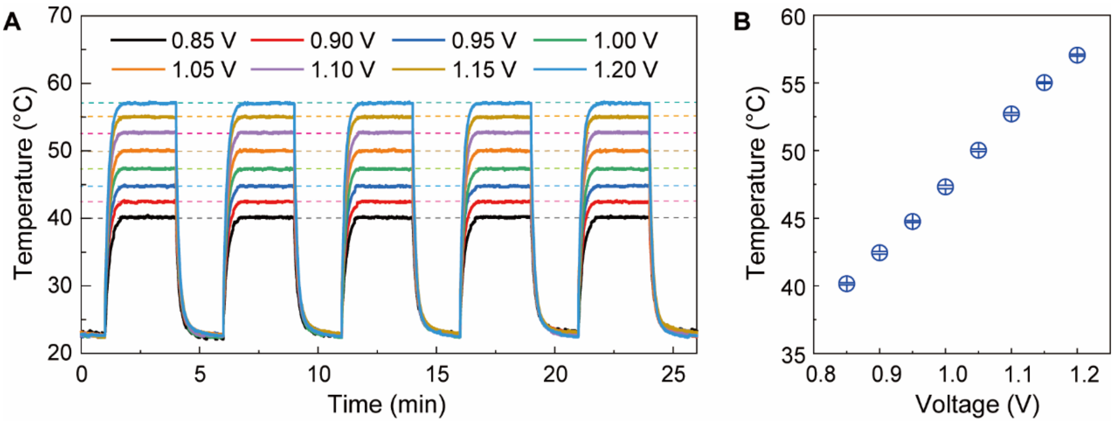

Figure S11. Temperature control of the electroresistive heater. (A) Time-dependent surface temperatures of the liquid metal-based serpentine mesh heaters with different supplied voltages. (B) Stabilized temperature *versus* voltage. The minor fluctuations within 0.5 °C at each voltage ensure the precise control over the surface temperature.

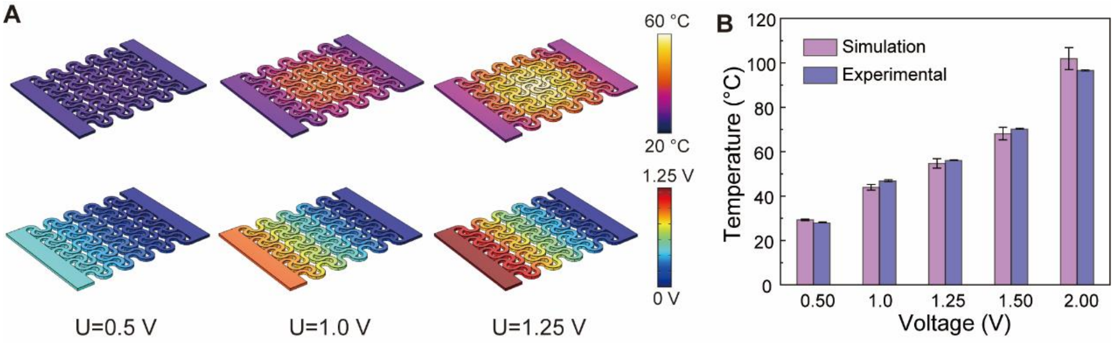

Figure S12. Finite element analysis of the electroresistive heater. (A) Surface temperature (top) and voltage (bottom) distributions on the serpentine mesh heater at different supplied voltages. (B) Comparison of simulated and measured temperature at different supplied voltages. The finite element simulation shows excellent consistency with the experiments. The three-dimensional finite element analysis was carried out by using COMSOL Multiphysics software. The TPU substrate is assigned with a thermal conductivity of 0.4 W m^{-1} K^{-1} and a negligible electrical conductivity. The EGaIn alloy adopts a known electrical conductivity of 3.4×10^4 S cm^{-1}. A refined mesh was used to ensure computation accuracy. Data are presented as mean ± SD of $n = 9$.

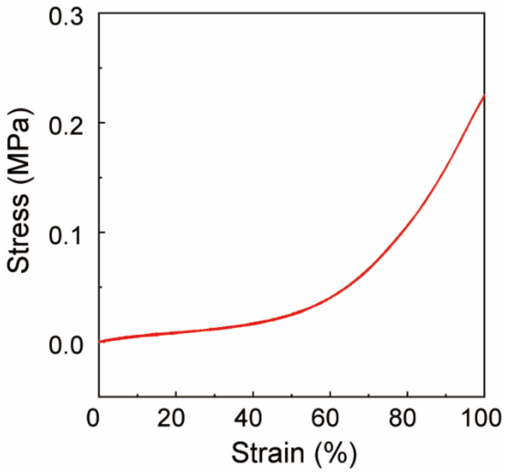

Figure S13. Uniaxial tensile stress-strain curve of compliant electroresistive heater. The effective modulus is determined as ~41.0 kPa.

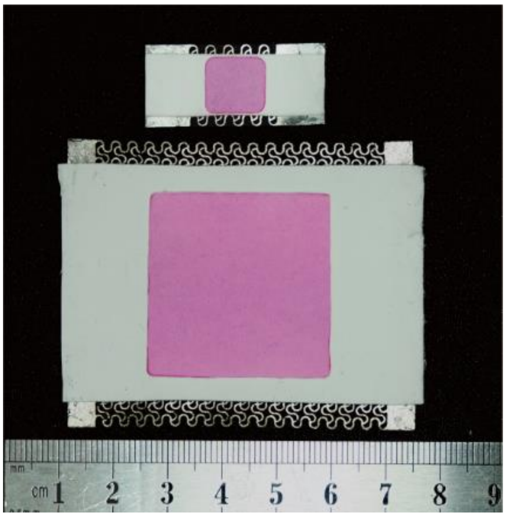

Figure S14. Stretchable electronic patches of different dimensions. A standard patch with 10 mm × 10 mm drug-loaded dressing is sufficient for small animal models (top). An enlarged patch design contains 33 mm × 33 mm drug-loaded dressing potentially suitable for large animal models/ human subjects (bottom).

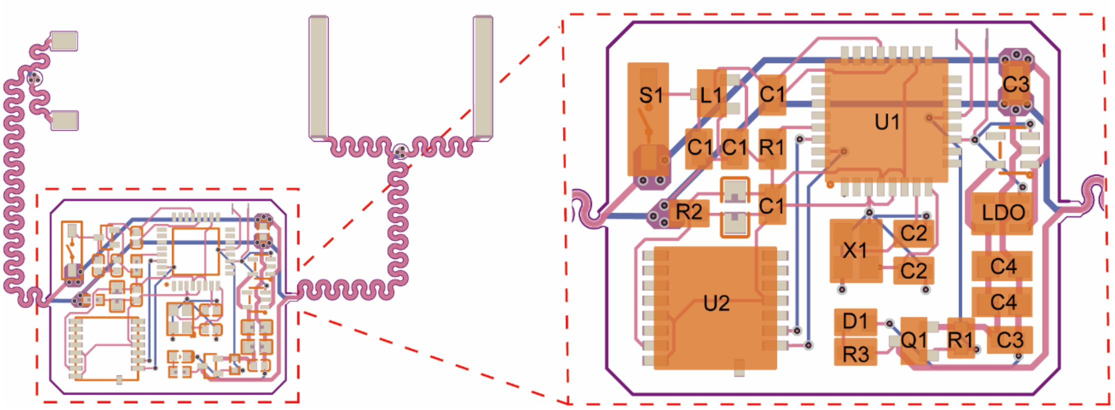

Figure S15. Schematic layout of the flexible integrated circuit board. The specifications of chip-scale components are summarized in Table S1.

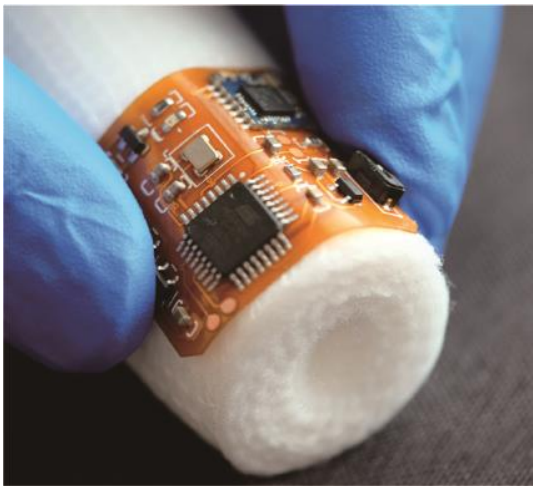

Figure S16. Optical image showing the flexibility of the integrated circuit board.

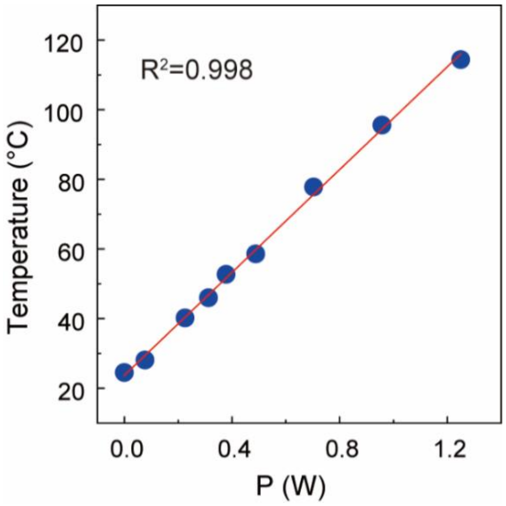

Figure S17. Temperature of the heater as a function of supplied electrical power. The temperature is proportional to the supplied electrical power. In the self-powered system, the PWM control effectively discretizes the maximal power supply of 3.35 W with a pre-scaler of 256. The corresponding temperature resolution is estimated to be better than 1 °C.

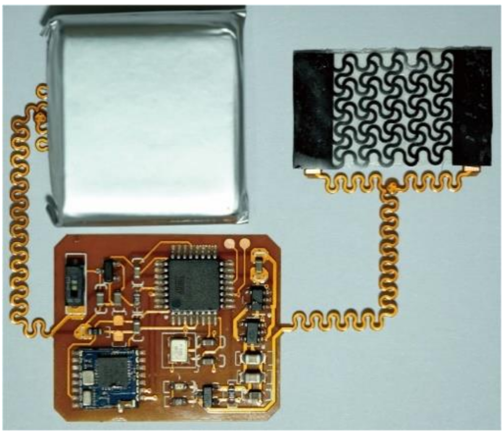

Figure S18. Optical images of an integrated electronic system customized for mice.

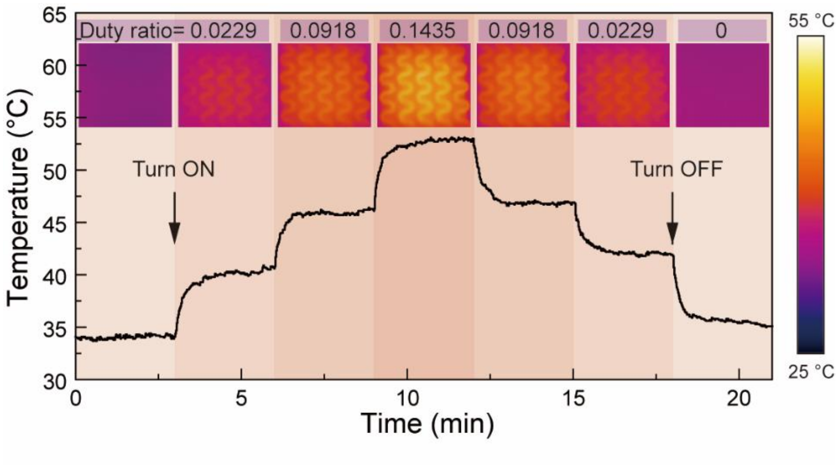

Figure S19. Surface temperature of the compliant heater at different duty ratios. Inset: Corresponding infrared camera images of the heater.

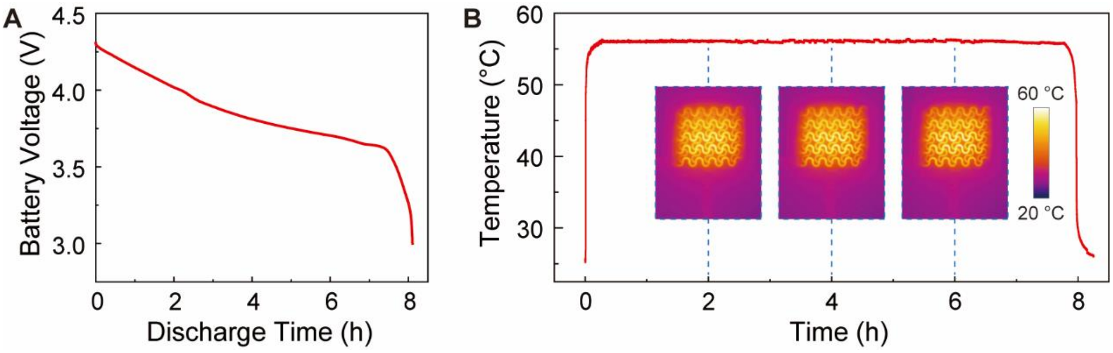

Figure S20. Long-term operation of the self-powered wearable electronic system. (A) Galvanostatic discharge profile of the lithium-ion battery at 1/8 C rate. The current is calibrated to sustain stable heating temperature at 55 °C of the system. (B) Temperature profile of the patch during continuous operation of the electronic system. The battery has extra capacity to support >90 thermal actuation cycles. In practice, the size of the battery can be further reduced to cut down the overall form factor of the system.

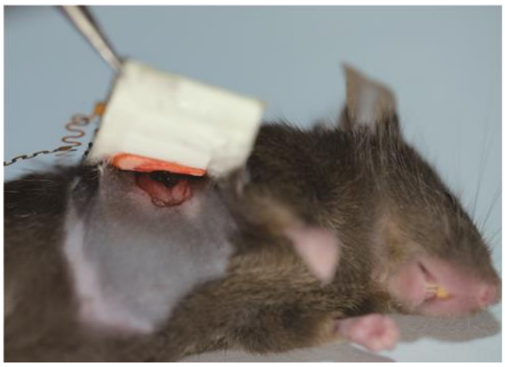

Figure S21. Optical image of a stretchable electronic patch attached to the surgical site of a postoperative mouse. The excellent mechanical deformability allows conformal and intimate interactions with the skin for efficient heat transfer and drug delivery.

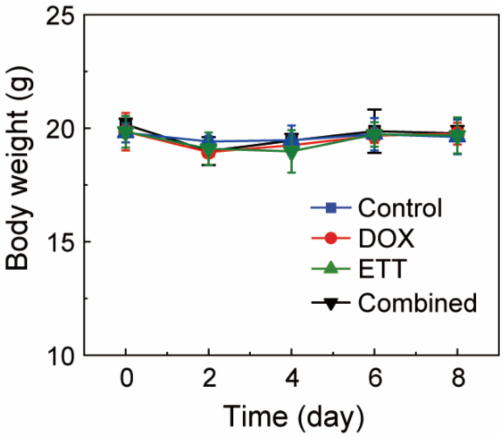

Figure S22. Changes in body weight of mice during the treatments. Data are presented as mean ± SD of n = 4.

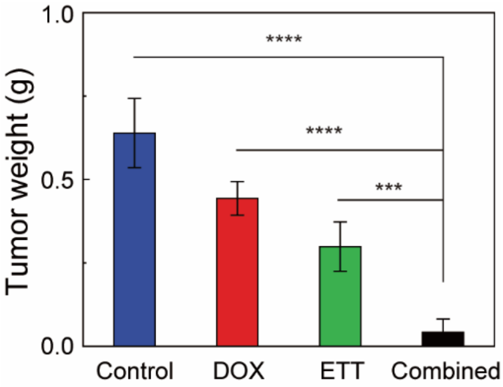

Figure S23. Weight of harvested tumors after the treatments. Data are presented as mean ± SD of n = 4. Statistical differences are determined by two tailed Student's t tests (*$p < 0.05$, **$p < 0.01$, ***$p < 0.001$, and ****$p < 0.0001$).

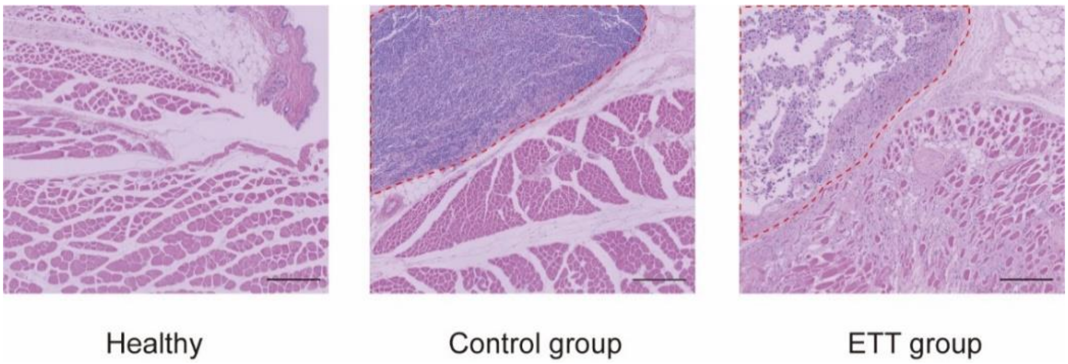

Figure S24. H&E histopathological analysis of healthy tissue (left), tumor region in the control group (middle), and tumor region in the ETT group (right). The healthy tissue comprises muscle fibers with peripheral nuclei and uniform sizes. In the control group, the tumor tissue exhibits a compact and dense pathogeny structure as encircled by the red dashed line. In contrast, ETT has a strong ablation effect on the tumor tissue after 8-day treatment. The surrounding tissue has the signatures of minor inflammation. The muscle fibers show a negligible reduction in thickness and a slight shrinkage along the longitudinal section. Scale bars: 200 μm.

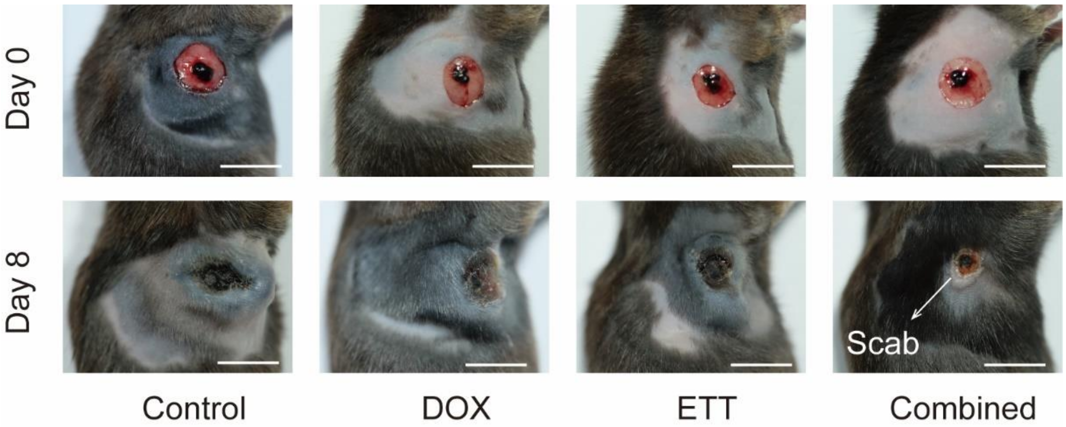

Figure S25. Optical images of postoperative wound sites acquired from different treatment groups. Scale bars: 10 mm.

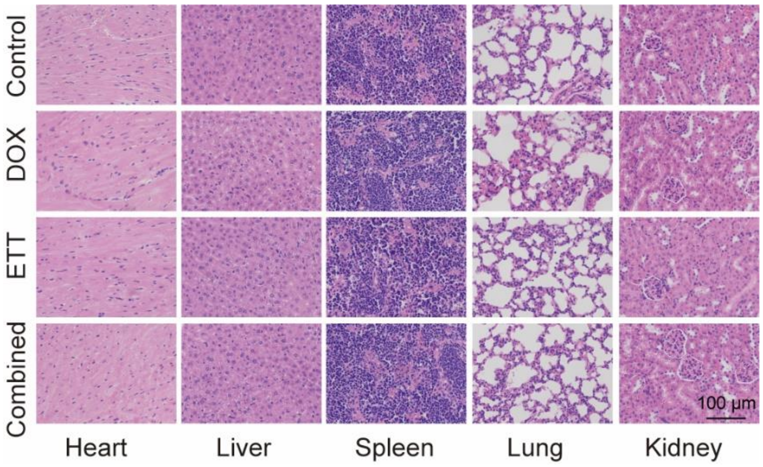

Figure S26. H&E histopathological analysis of major organ tissues after the treatments. In all experimental groups, the lack of significant tissue damage demonstrates the low toxicity and safety of the protocols.

Supporting Table

Table S1. Chip information of the flexible integrated circuit.

Label	Type	Value	Part number	Manufacturer
**U1**	MCU	NA	ATMEGA328P-AU	Microchip Technology
**U2**	Bluetooth Module	NA	RF-BM-4404B4	Shenzhen RF-star Technology
**LDO**	LDO	NA	TLV75733PDBVR	Texas Instruments Incorporated
**Q1**	MOSFET	NA	YJL3401A	Yangzhou Yangjie Electronic Technology
**S1**	Switch	NA	CHS-01TA1	Nidec Corporation
**D1**	Red LED	NA	EL0603/R6C-A01/DT	Everlight Electronics
**X1**	ECO	NA	3225-8.00-10-10-10/A	Suzhou Liming Electronic Co.
**R1**	Resistor	10 kΩ	ERJUP3J103V	Panasonic Industry
**R2**	Resistor	0 Ω	ERJS030R00V	Panasonic Industry
**R3**	Resistor	3.3 kΩ	ERA3AEB332V	Panasonic Industry
**C1**	Inductor	100 nF	GCM188R71H104JA57D	Murata Manufacturing
**C2**	Inductor	16 pF	GRM1885C1H160JA01D	Murata Manufacturing
**C3**	Inductor	22 µF	CL10A226MQ8NRNC	Samsung Electro-Mechanics
**C4**	Inductor	100 µF	GRM21BR60J107ME15L	Murata Manufacturing
**L1**	LDO	NA	SC662K-3.3V	Fuman Microelectronics Group

MCU: microcontroller unit

MOSFET: metal-oxide-semiconductor field-effect transistor

LDO: low-dropout voltage regulator

ECO: External Crystal Oscillator

Supporting Videos

Video S1. Integrated electronic system operated on a freely moving mouse. Stretchable electronic patch is attached to postoperative wound site in the axilla to enable wearable cancer therapy.

Video S2. Integrated electronic system mounted on a human forearm for remotely controlled thermal actuation. The entire system is of compact design to allow convenient carry on the forearm. All control parameters are set on the smartphone and wirelessly sent to the microcontroller. The surface temperature of the electronic turned off after set duration.

Video S3. Stretchable electronic patch with the conformal attachment to the wrist under continuous hand movements.
